# [Retracted] Knockdown of SNHG16 suppresses the proliferation and induces the apoptosis of leukemia cells via miR-193a-5p/CDK8

**DOI:** 10.3892/ijmm.2024.5389

**Published:** 2024-06-17

**Authors:** Meihua Piao, Li Zhang

Int J Mol Med 46: 1175-1185, 2020; DOI: 10.3892/ijmm.2020.4671

Following the publication of this paper, it was drawn to the Editor's attention by a concerned reader that certain of the colony formation assay data shown in Fig. 7A on p. 1183 were strikingly similar to data appearing in different form in the following article written by different authors at different research institutes that had already been published prior to its date of submission: Lou L, Chen G, Zhong B and Liu F: *Lycium barbarum* polysaccharide induced apoptosis and inhibited proliferation in infantile hemangioma endothelial cells via down-regulation of PI3K/AKT signaling pathway. Biosci Rep 39: BSR20191182, 2019. In addition, possible anomalies were noted regarding the appearance of the western blots in the paper. Owing to the fact that the contentious data in the above article had already been published prior to its submission to *International Journal of Molecular Medicine*, the Editor has decided that this paper should be retracted from the Journal. The authors were asked for an explanation to account for these concerns, but the Editorial Office did not receive a reply. The Editor apologizes to the readership for any inconvenience caused.

